# The Clinical Impact of the Introduction of a Robot‐Assisted Program in a Specialized Hernia Center: A Propensity Score Matched Cohort Study on Short‐Term Outcomes

**DOI:** 10.1002/wjs.12477

**Published:** 2025-01-15

**Authors:** Mads Marckmann, Mette Willaume Christoffersen, Nadia A. Henriksen, Kristian S. Kiim

**Affiliations:** ^1^ Digestive Disease Center Bispebjerg Hospital Copenhagen Denmark; ^2^ Department of Surgery Zealand University Hospital Koege Denmark; ^3^ Department of Gastroenterology and Hepatology Surgical Section Copenhagen University Hospital Herlev‐Gentofte Copenhagen Denmark; ^4^ Department of Transplantation and Surgery Rigshospitalet Copenhagen Denmark

**Keywords:** hernia repair, length of stay, readmission, reoperation, robot‐assisted surgery

## Abstract

**Background:**

The role of robot‐assisted approach in hernia surgery remains controversial due to high procedural costs and the proposed equal outcomes compared with open surgery. In this study, we report the 30‐day results of the introduction of robot‐assisted approach in a specialized regional ventral hernia repair center.

**Methods:**

This was a retrospective single‐center cohort study including patients undergoing either robot‐assisted or open ventral hernia repair from 2017 to 2022. Patients undergoing either approach were matched by propensity scores in a 1:2 ratio on the variables age, type of hernia (primary/incisional), and horizontal fascial defect size to reduce bias risk. Multivariable logistic regression on outcomes length of stay, reoperation, and readmission was performed.

**Results:**

A total of 109 patients undergoing robot‐assisted repair were compared to 229 undergoing open repair. Overall, 61.2% were patients had incisional hernia. Mean hernia defect size was 4.9 × 6.5 cm (horizontal × vertical). The mean length of stay was shorter after robot‐assisted repair (0.1 vs. 1.9 days, *p* < 0.001) as was the incidence of readmission (3.7% vs. 17.0%, *p* < 0.001). The incidence of reoperation was tangentially significantly lower after robot‐assisted repair (0.9% vs. 6.6%, *p* = 0.045); however, the estimate was significant after adjusting for confounders (OR 0.11, CI 0.01–0.89, *p* = 0.038).

**Conclusions:**

Length of stay and readmission rates were significantly decreased after the introduction of a robot‐assisted approach for ventral hernia repair.

## Graphical abstract

Hypervascular ICC, quantified by CT vascularity, represents a favorable prognosis after surgical resection, and there is a possibility of omitting lymphadenectomy in this subgroup.

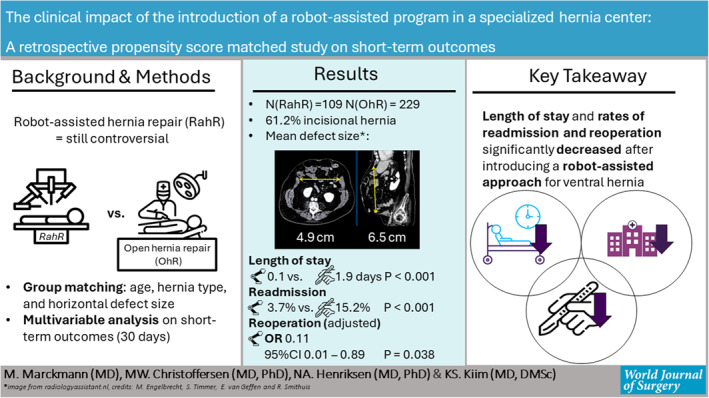

## 1 Introduction

The impact of robot‐assisted hernia repair on surgical outcomes remains controversial. Studies have showed promising results after robot‐assisted repair in terms of decreased length of stay (LOS), less postoperative pain, and less wound morbidity, whereas disadvantages are longer operation times and higher procedural costs [1, 2, 3, 4]. However, recent studies have suggested that the longer operation times and higher procedural costs are offset by fewer systemic complications and lower short‐term morbidity [5, 6, 7, 8, 9]. Robot‐assisted hernia repair has been established as reproducible, safe, and feasible with the use of the enhanced‐view totally extraperitoneal technique (eTEP), transabdominal retromuscular umbilical prosthetic hernia repair (TARUP), and RoboTAR (robotic transversus abdominis release) in patients with ventral hernia that would otherwise often be offered an open procedure [2, 4, 6, 10].

Furthermore, a recent study described a high rate of same‐day discharge with low levels of postoperative pain, whereas another study showed lower rates of conversion to open repair and fewer early operations for complications in patients undergoing robot‐assisted repair compared to laparoscopic and open approaches [11, 12]. Patient‐reported outcome measures (PROMs), for example, quality of life, reportedly also improved in favor of the robot‐assisted approach in comparison to laparoscopic and open approaches [3, 13]. Opposingly, randomized trials reported higher costs and equal outcomes after robot‐assisted repair compared with laparoscopic minimally invasive techniques [14, 15, 16, 17].

In summary, the current available data regarding robot‐assisted hernia repair remain heterogenic with evidence of varying quality. The aim of this study was to investigate the short‐term outcomes in a matched cohort after the introduction of a robot‐assisted program in a specialized hernia center.

## 2 Material and Methods

### 2.1 Study Design and Setting

This was a retrospective single‐center cohort study including consecutive patients undergoing open or robot‐assisted ventral hernia repair from January 1, 2017 to December 31, 2022. Robot‐assisted approach was implemented March 1, 2021 at a university hospital. Data for the open operations were collected retrospectively, whereas the robot‐assisted cases were registered prospectively with consecutive follow‐ups 30 days postoperatively for each participant.

### 2.2 Participants

All patients undergoing either open or robot‐assisted ventral primary or incisional hernia repair during the study period were eligible for inclusion. Furthermore, inclusion criteria were age ≥ 18 years and ability to provide informed consent. Patients admitted for emergency hernia repair were excluded. Cases were ascertained as patients undergoing robot‐assisted surgery and controls as open procedures, referring to the case group as the experimental treatment evaluated. This was done by the preference of the surgeons. Cases and controls were matched by propensity scores in a 1:2 ratio on the variables age, type of hernia (primary/ventral), and horizontal fascial defect size.

The surgical procedures performed varied to some extent; however, the general approach and the robot‐assisted procedures have previously been described in detail [18, 19]. Parts of the results of this cohort have previously been reported [12].

### 2.3 Variables

Preoperative baseline data included age, sex (binarily categorized with referral to the sex assigned by the patient's social security number at the time of inclusion), smoking status (yes/no), American Society of Anesthesiologists (ASA) score, hernia type (primary/incisional), and body mass index (BMI). Intraoperative variables were defect size measures (horizontal and vertical), surgical approach (transabdominal preperitoneal [TAPP], bilateral transversus abdominis release [RoboTAR], transabdominal retromuscular umbilical prosthetic repair [TARUP], enhanced‐view totally extraperitoneal [eTEP], open, retromuscular), mesh placement (preperitoneal, retromuscular, onlay, and intraperitoneal), mesh name, and whether or not component separation was performed. Outcomes assessed 30 days postoperatively included length of stay (LOS), readmission, and reoperation.

### 2.4 Data Sources/Measurement

All baseline data were collected preoperatively in the outpatient clinic. Intraoperative variables were registered by the surgeon in charge of the procedure. The three outcomes were registered in the electronical patient chart, and data were extracted 30 days postoperatively. Assessment methods for all variables were comparable between both case and control groups.

### 2.5 Bias

The nature of this study's methodological approach represents real‐life data with a risk of selection bias. Propensity score matching was used to reduce heterogeneity, balance treatment groups, and eventually limit bias in the estimated treatment effect.

### 2.6 Quantitative Variables

To enable comparability with other studies, age, BMI, fascial defect size, and length of stay were all categorized.

### 2.7 Statistical Methods

Cases and controls were matched by the propensity score in a 1:2 ratio according to the variables age, type of hernia (primary/incisional), and horizontal fascial defect size. The matching was performed using the nearest neighbor method and a 0.05 caliper. Continuous variables were given as mean (standard deviation, SD) or median (interquartile range, IQR) according to the normality of data and were compared across groups by either Student's *t*‐test or the Mann–Whitney U test. Categorical variables were reported as *n* (%) and compared across groups by the chi‐square test. Multivariable logistic regression was performed to disclose potential confounders. Variables adjusted for included age, sex, horizontal defect size, surgical approach (open vs. robot‐assisted), type of hernia (primary/incisional), and BMI, which were selected based on the literature.

The data analysis was performed using R software version 4.0.2 (R Foundation for Statistical Computing, Vienna, Austria). This study was approved by the Danish Data Protection Agency (ref. P‐2021‐58), and all patients gave consent to the chart review. The study was reported according to the STROBE guidelines [20].

## 3 Results

A total of 338 patients were included in the study of which 109 (32.2%) underwent robot‐assisted ventral hernia repair (cases) and 229 open ventral hernia repairs (controls). Across the two groups, the variables age, sex, hernia defect size, and hernia type were comparable. A significantly higher rate of patients in the robot group were active smokers compared with the open group (33.0% vs. 12.2% *p* < 0.001). Further, there were more patients with a BMI > 35 kg/m^2^ in the robot group (29 (26.6%) versus 29 (12.8%) *p* = 0.027). A total of 11 different meshes were used across the entire cohort, with a significant difference between the robot‐assisted group and the open group (*p* < 0.001). For patients undergoing robot‐assisted repair, the TARUP procedure was most frequently used (71 (65.1%)) along with RoboTAR (21 (19.3%)). In 102 (93.6%) of the patients undergoing robot‐assisted repair, the mesh was placed in the retromuscular plane, whereas mesh placement varied to a greater extent in patients undergoing open repair (Table 1). Two robot‐assisted procedures required conversion to open surgery: one because of intraoperative respiratory complications and one due to technical inability to excise an earlier applied mesh without skin perforation.

**TABLE 1:** Preoperative, intraoperative, and postoperative data comparing patients who underwent robot‐assisted ventral hernia repair with patients who underwent open ventral hernia repair.

			Robot‐assisted approach (*n* = 109)	Open repair (*n* = 229)	Total (*n* = 338)	*p*
PREOPERATIVE	Age	< 45	18 (16.5)	35 (15.3)	53 (15.7)	0.989
		45–60	41 (37.6)	85 (37.1)	126 (37.3)	
		> 60–75	36 (33.0)	78 (34.1)	114 (33.7)	
		> 75	14 (12.8)	31 (13.5)	45 (13.3)	
	Sex	Female	44 (40.4)	99 (43.2)	143 (42.3)	0.704
	Smoking	Yes	36 (33.0)	28 (12.2)	64 (18.9)	< 0.001
	ASA	I	30 (27.5)	61 (26.6)	91 (26.9)	0.139
		II	55 (50.5)	136 (59.4)	191 (56.5)	
		III	24 (22.0)	32 (14.0)	56 (16.6)	
	Body Mass Index	Mean [sd]	31.4 [5.5]	29.1 [5.5]	29.8 [5.6]	< 0.001
		Missing	0	3	3	
	*Categorized*	< 30	52 (47.7)	136 (60.2)	188 (56.1)	0.006
		30–35	28 (25.7)	61 (27.0)	89 (26.6)	
		> 35	29 (26.6)	29 (12.8)	58 (17.3)	
		Missing	0	3	3	
INTRAOPERATIVE	Vertical defect size, cm	Mean (sd)	6.6 (5.4)	6 (5.4)	6.2 (5.4)	0.336
	Horizontal defect size, cm	Mean (sd)	4.8 (3.2)	4.9 (3.5)	4.9 (3.4)	0.799
	*Categorized*	< 4	67 (61.5)	139 (60.7)	206 (60.9)	0.771
		4–8	27 (24.8)	52 (22.7)	79 (23.4)	
		> 8	15 (13.8)	38 (16.6)	53 (15.7)	
	Approach	TAPP	7 (6.4)	0 (0.0)	7 (2.1)	< 0.001
		RoboTAR	21 (19.3)	0 (0.0)	21 (6.2)	
		TARUP	71 (65.1)	0 (0.0)	71 (21.0)	
		eTEP	9 (8.3)	0 (0.0)	9 (2.7)	
		Open	0 (0.0)	229 (100.0)	229 (67.8)	
		Retromuscular	1 (0.9)	0 (0.0)	1 (0.3)	
	Mesh placement	Preperitoneal	7 (6.4)	47 (20.5)	54 (16.0)	< 0.001
		Onlay	0 (0.0)	33 (14.4)	33 (9.8)	
		Retromuscular/sublay	102 (93.6)	115 (50.2)	217 (64.2)	
		Intraperitoneal	0 (0.0)	34 (14.8)	34 (10.1)	
	Component separation	No	79 (72.5)	201 (87.8)	280 (82.8)	< 0.001
		Bilateral	19 (17.4)	28 (12.2)	47 (13.9)	
		Unilateral	11 (10.1)	0 (0.0)	11 (3.3)	
	*Type*	None	79 (72.5)	200 (87.3)	280 (82.8)	< 0.001
		TAR	30 (27.5)	19 (8.3)	48 (14.2)	
		ACS	0 (0.0)	10 (4.4)	10 (3.0)	
	Type of hernia	Primary	43 (39.4)	86 (37.6)	129 (38.2)	0.829
		Incisional	66 (60.6)	143 (62.4)	209 (61.8)	
	Mesh name	Parietex Progrip	91 (83.5)	101 (44.1)	192 (56.8)	< 0.001
		Softmesh	18 (16.5)	16 (7.0)	34 (10.0)	
		Ventralex ST, BARD	0 (0.0)	66 (28.8)	66 (19.5)	
		Parietene, Covidien	0 (0.0)	4 (1.7)	4 (1.2)	
		Versatex	0 (0.0)	31 (13.5)	31 (9.2)	
		Ventrio Hernia ST, BARD	0 (0.0)	2 (0.9)	2 (0.6)	
		Ventralight ST, BARD	0 (0.0)	2 (0.9)	2 (0.6)	
		Adhesix, BARD	0 (0.0)	4 (1.7)	4 (1.2)	
		Vypro, Ethicon	0 (0.0)	1 (0.4)	1 (0.3)	
		Galmeh Light	0 (0.0)	1 (0.4)	1 (0.3)	
		Parietene Composite	0 (0.0)	1 (0.4)	1 (0.3)	
POSTOPERATIVE	Readmission	Yes	4 (3.7)	39 (17.0)	43 (12.7)	0.001
	Reoperation	Yes	1 (0.9)	15 (6.6)	16 (4.7)	0.045
	Length‐of‐stay, days	Mean [range]	0 [0, 3]	2.1 [0, 19]	1.4 [ 0, 19]	< 0.001
	*Categorized*	0	108 (99.1)	123 (54.7)	231 (69.2)	< 0.001
		1	1 (0.9)	102 (45.3)	103 (30.8)	
		Missing	0	4	4	

A significant higher number of patients in the open group were readmitted and reoperated compared to the robot group (17% vs. 3.7% *p* = 0.001 and 6.6% vs. 0.9% *p* = 0.045, respectively). All patients but one in the case group were discharged on the first postoperative day, whereas significantly more in the control group required more than 1 day in the hospital postoperatively (45.3% vs. 0.9% *p* < 0.001). Component separation was performed more frequently during robot‐assisted repair (27.5% vs. 12.2% *p* < 0.001) including *transverse abdominis release* as the preferred technique (27.5% vs. 8.3% *p* < 0.001). For patients undergoing either uni‐ or bilateral component separation, the median length of stay was significantly decreased after robot‐assisted approach (0 vs. 4 days, *p* < 0.001).

After multivariable logistic regression analysis of factors associated with reoperation within 30 days after surgery, results showed a significantly lower risk when undergoing robot‐assisted ventral hernia repair compared to open (OR 0.11 95% CI 0.01; 0.89 *p* = 0.038). Male sex and horizontal defect size between 4 and 8 cm showed an independently increased risk of reoperation (OR 4.84 95% CI 1.25; 18.79 *p* = 0.023 and OR 5.08 95% CI 1.28; 20.08 *p* = 0.021).

Logistic regression analysis of factors associated with length of stay > 1 day showed that robot‐assisted repair decreased the risk of overnight stay (OR 0.00 95% CI 0.00; 0.03 *p* < 0.001). Horizontal defect size > 4 cm increased the risk of prolonged LOS, with a defect of > 8 cm posing the highest risk (OR 13.18 95% CI 4.56; 38.12 *p* < 0.001). Primary ventral hernia repair was independently associated with a decreased risk of overnight stay in hospitals compared to incisional hernia repair when adjusting for the chosen confounders (OR 0.09 95% CI 0.03; 0.22 *p* < 0.001). Finally, patients undergoing robot‐assisted repair had a significantly lower risk of readmission within 30 days compared to the group undergoing open surgery (OR 0.17 95% CI 0.06; 0.51 *p* = 0.001), whereas a BMI > 35 kg/m^2^ was independently associated with a higher risk of readmission; however, the signal was not statistically significant (OR 2.27 95% CI 0.93; 5.51 *p* = 0.070). Tables 2, 3, 4 show the results from the multivariable analyses with regards to reoperation, length of stay in hospital, and readmission, respectively.

**TABLE 2:** Multivariable logistic regression analysis on factors associated with reoperation within 30 days after ventral hernia repair.

		Odds ratio (OR)	95% CI	*p*
Age	< 45	Ref		
	45–60	0.68	[0.11; 4.02]	0.668
	> 60–75	0.41	[0.07; 2.59]	0.345
	> 75	0.36	[0.03; 4.73]	0.435
Sex	Female	Ref		
	Male	4.84	[1.25; 18.79]	0.023
Horizontal defect, cm	< 4	Ref		
	4–8	5.08	[1.28; 20.08]	0.021
	> 8	1.99	[0.38; 10.36]	0.414
Robot‐assisted approach	Yes	0.11	[0.01; 0.89]	0.038
Type of hernia	Incisional	Ref		
	Primary	0.12	[0.01; 1.05]	0.056
Body Mass Index	< 30	Ref		
	30–35	2.57	[0.73; 9.09]	0.142
	> 35	2.95	[0.69; 12.60]	0.145

**TABLE 3:** Multivariable logistic regression analysis on factors associated with length of stay > 1 day after ventral hernia repair.

		Odds ratio (OR)	95% CI	*p*
Age	< 45	Ref		
	45–60	3.66	[0.98; 13.65]	0.053
	> 60–75	4.17	[1.12; 15.53]	0.033
	> 75	2.48	[0.54; 11.38]	0.242
Sex	Female	Ref		
	Male	0.76	[0.36; 1.59]	0.467
Horizontal defect, cm	< 4	Ref		
	4–8	8.62	[3.51; 21.16]	< 0.001
	> 8	13.18	[4.56; 38.12]	< 0.001
Robot‐assisted approach	Yes	0.00	[0.00; 0.03]	< 0.001
Type of hernia	Incisional	Ref		
	Primary	0.09	[0.03; 0.22]	< 0.001
Body Mass Index	< 30	Ref		
	30–35	1.54	[0.67; 3.52]	0.309
	> 35	2.05	[0.65; 6.46]	0.220

**TABLE 4:** Multivariable logistic regression analysis on factors associated with readmission within 30 days after ventral hernia repair.

		Odds ratio (OR)	95% CI	*p*
Age	< 45	Ref		
	45–60	1.90	[0.58; 6.21]	0.289
	> 60–75	1.45	[0.42; 4.96]	0.555
	> 75	1.93	[0.17; 5.13]	0.940
Sex	Female	Ref		
	Male	0.82	[0.41; 1.64]	0.578
Horizontal defect, cm	< 4	Ref		
	4–8	1.25	[0.53; 2.98]	0.647
	> 8	1.25	[0.48; 3.25]	0.608
Robot‐assisted approach	Yes	0.17	[0.06; 0.51]	0.001
Type of hernia	Primary	Ref		
	Incisional	1.31	[0.58; 2.94]	0.520
Body Mass Index	< 30	Ref		
	30–35	1.05	[0.46; 2.38]	0.906
	> 35	2.27	[0.93; 5.51]	0.070

## 4 Discussion

In the current study, the introduction of a robot‐assisted ventral hernia repair program was significantly associated with a decreased risk of reoperation 30 days postoperatively. Further, the incidence of prolonged length of stay as well as readmission was reduced after the introduction of the robot‐assisted program.

The reduced risk of reoperation may be explained by a lower incidence of wound complications after robot‐assisted repair [7, 21, 22, 23]. Martin‐Del Campo et al. compared patients undergoing open and robot‐assisted transversus abdominis release (rTAR) and did not register any surgical site infections (SSI) in the rTAR group opposed to 6.6% in the open; however, the difference was not statistically significant, most likely due to type II errors [7]. This explanation is also supported by the fact that minimally invasive techniques in general reduce the incidence of SSI in patients undergoing ventral hernia repair [24].

A key finding of the current study is that only one patient in the robot group required more than 1 day in the hospital as opposed to nearly half of the patients in the open group. Increasing horizontal defect size was associated with longer hospital stay, which echoes earlier reports, probably due to the greater dissection required for defect closure and longer duration of surgery [25]. Shorter length of stay is one of the most established favors of robot‐assisted surgery, and the findings of this study also add support to this upside of robot‐assisted surgery [1, 3, 5, 6, 7, 9, 26, 27]. Notably, the length of stay was greatly reduced in patients undergoing component separation using a robot‐assisted approach, reflecting previous reports [22]. This difference was present even though patients undergoing open repair were subjected to a strict enhanced recovery protocol, indicating a high impact of robot‐assisted approach in patients with large ventral hernia [28].

The incidence of readmission was also significantly lower for patients who underwent robot‐assisted surgery. This correlates with earlier reports including a large population‐based study comparing both laparoscopic, robotic, and open repair [5, 26]. Again, this is readily explained by the unequal distribution of surgical site occurrences, but also pain levels should be considered, as these have been reported as higher for patients undergoing open surgery compared to both laparoscopic and robot‐assisted approaches, and is a well‐known reason for hospitalization [29, 30, 31].

At our department, the introduction of robot‐assisted hernia repair enabled a transformative process from open surgery, requiring lengthy postoperative hospital stay, to an outpatient setting with planned discharge on the same day of surgery, releasing significant amounts of resources including higher capacity in the inpatient ward. This gives rise to economic considerations; we did not evaluate the possible differences in procedural costs, which would have been interesting since an often highlighted disadvantage of robot‐assisted repair is the higher procedure‐related costs compared to conventional approaches, that is, laparoscopic or open [14, 15, 32]. Importantly, there were no risks of economical or insurance‐related bias concerning patient group assignment as the study's hospital is a public health institution in Denmark, where all costs are covered through social security.

This study reflects a steep learning curve by the direct clinical impact of implementing the approach from day one and onwards. As evident, transitioning from open surgery to offering patients robot‐assisted hernia repair led to an immediate effect on our clinical setup with convincing feasibility regarding both the gained ability of same‐day discharge but also the positive side effect of setting free resources at our hospital. The robot‐assisted technique was new, and one should expect a learning curve, yet outcomes were still evidently better compared to the well‐established open approach. These results support earlier results of the robot‐assisted approach as safe and reproducible [1, 4, 10, 33]. The robot‐assisted procedures in the current study were performed by a total of three different surgeons who all underwent formal training prior to the introduction of a robotic approach at our institution. We strongly believe that focused training and a high yearly procedural volume are key to improving outcomes when introducing new surgical techniques, and as such, we think this serves as one explanation of the results of the current study.

The robot‐assisted approach predominantly involved mesh placement in the retromuscular position, which illustrates another advantage of robot‐assisted repair: easier access to the retromuscular or preperitoneal plane and mesh placement. This mesh localization is preferred and includes lower risk of wound morbidity, less need for mesh fixation, and lower risk of postoperative chronic pain compared to other mesh localizations, for example, intraperitoneal onlay mesh (IPOM) [34, 35, 36]. Also, for retromuscular mesh placement in hernias horizontally incised, a TAR procedure is often necessary, whereas intraperitoneal or onlay mesh placement in an open surgery setup does not obligate TAR. These conditions readily explain the difference in the frequency of component separation between the two groups found in the current study.

There were significantly more obese patients and smokers in the robot‐assisted group, a fact that could raise concern as these factors pose a risk for wound complications in open hernia surgery [37]. However, our results suggest that higher weight and active smoking are not inherent barriers for robot‐assisted hernia repair, which correlates with earlier reports on minimally invasive surgery [18]. We also found that higher BMI did not increase the risk of short‐term reoperation, longer stay in hospital, or readmission within 30 days after ventral hernia repair (Table 4).

Lastly, male sex was independently associated with a higher risk for reoperation within 30 days (OR 4.76). From our data, there is no obvious explanation to this. One study found that females had a higher risk for inguinal hernia recurrence, whereas another one found female sex as a risk factor for reoperation after general abdominal surgery [38, 39].

Limitations of this study include the single‐center setup and few number of surgeons in charge, which compromises external validity. Also, as the robot‐assisted program was implemented, the first patients offered this approach were selected to be mostly unadvanced and smaller hernias with subsequent increased difficulty, a temporal fact not addressed in the data analyses. Surgical site occurrences, for example, seroma formation, which were not treated surgically, were not registered for this study, which could have been very interesting to evaluate as lower incidences might be expected with minimally invasive techniques. SSOs requiring procedural intervention, however, were weighed to have higher clinical impact. Lastly, patient‐reported outcome measures are highly relevant when assessing the effect of robot‐assisted surgery, but unfortunately, these data were not accessible for this study design; however, earlier results showed an increased quality of life when applying a robot‐assisted approach [13].

In conclusion, the findings of the current study suggest that compared to open hernia repair, the introduction of robot‐assisted hernia surgery in an elective setting is unharmful, leads to fewer readmissions, reduced risk of reoperation, and a high rate of same‐day surgery favoring both patients and health care resources. Further data and larger studies on this subject, including comparative costs analyses, are needed to adamantly establish and secure the role of robots in hernia surgery.

## Author Contributions

**Mads Marckmann:** data curation, visualization, writing–original draft, writing–review & editing. **Mette Willaume Christoffersen:** project administration, writing–review & editing. **Nadia A. Henriksen:** writing–review & editing. **Kristian S. Kiim:** conceptualization, data curation, formal analysis, investigation, methodology, project administration, resources, software, supervision, validation, writing–review & editing.

## Consent

This study was approved by the Danish Data Protection Agency (ref. P‐2021‐58), and all patients gave consent to chart review.

## Conflicts of Interest

M.M. and M.W.C. have nothing to declare. N.A.H. has received speaker fees from Medtronic and Gore. N.A.H. was a member of the Robotic Hernia Advisory Board for Hugo RAS, Medtronic. K.S.K. has received speaker fees from Medtronic, Intuitive Surgical, ConMed, and BD. K.S.K. received research grants from Intuitive Surgical.
